# Prevalence and factors associated with acute kidney injury in children presenting to the emergency department with a first tonic–clonic seizure: an exploratory study

**DOI:** 10.1007/s00431-026-07079-y

**Published:** 2026-05-22

**Authors:** Pierluigi Marzuillo, Manuela Cerrone, Anna Di Sessa, Paola Tirelli, Giulio Rivetti, Giusy Capasso, Rosa Melone, Emanuele Miraglia del Giudice, Stefano Guarino, Felice Nunziata

**Affiliations:** 1 Department of Woman, Child and of General and Specialized Surgery, Università Degli Studi Della Campania “Luigi Vanvitelli”, Via Luigi De Crecchio 2, 80138 Naples, Italy; 2 Department of Pediatric Subspecialties, Nephrology and Dialysis Unit, Children’s Hospital Bambino Gesù, IRCCS, Rome, Italy; 3 Department of Pediatrics, AORN Sant’Anna E San Sebastiano, Via Ferdinando Palasciano, 81100 Caserta, Italy

**Keywords:** Seizures, Febrile seizures, Acute kidney injury, Children, Ibuprofen, C-reactive protein

## Abstract

We aimed to determine the prevalence of acute kidney injury (AKI) and to identify factors associated with its development in children presenting with a first febrile or non-febrile tonic–clonic seizure. We conducted an exploratory retrospective study including children (< 18 years) presenting to the emergency department for a first episode of febrile or non-febrile tonic–clonic seizure between January 2021 and July 2025. AKI was defined according to KDIGO serum creatinine criteria, using back-calculating methods to estimate baseline creatinine. Clinical, laboratory, and therapeutic variables were collected. Logistic regression analyses were performed to identify factors associated with AKI. A total of 190 children (mean age 4.2 ± 3.7 years) were included; 112 (58.9%) presented with febrile seizures. AKI occurred in 23 patients (12.1%), with similar prevalence in febrile and non-febrile seizures (12.5% vs. 11.5%; *p* = 0.84). No patient required renal replacement therapy. Among children with febrile seizures, being born small for gestational age (SGA), elevated C-reactive protein (CRP) levels, and prior use of combined paracetamol and ibuprofen were independently associated with AKI. In the non-febrile seizure subgroup, longer seizure duration and higher CRP levels were associated with AKI in univariate analysis, but only CRP remained independently associated in multivariable model.

*Conclusion*: AKI occurs in approximately 12% of children presenting with a first tonic–clonic seizure. Elevated CRP levels were associated with AKI in both febrile and non-febrile seizures, while SGA status and combined use of antipyretics were associated with AKI among children with febrile seizures. Given the limited number of events, these findings should be interpreted as exploratory.

**Table:** 

**What is Known:** •* Acute kidney injury (AKI) has been anecdotally reported in patients with status epilepticus, likely related to rhabdomyolysis-induced nephropathy.* • *No studies have systematically investigated this association in either adults or children.* **What is New:** • *Approximately 12% of children presenting with a fi rst febrile or non-febrile tonic–clonic seizure develop acute kidneyinjury (AKI).* • *Elevated C-reactive protein (CRP) levels are independently associated with AKI in both febrile and non-febrileseizures, whereas small-for-gestational-age status and prior combined use of paracetamol and ibuprofen areadditional factors associated with AKI in children with febrile seizures.*

**Supplementary Information:**

The online version contains supplementary material available at 10.1007/s00431-026-07079-y.

## Introduction

Acute seizures are a frequent cause of pediatric emergency department visits [1, 2]. They affect approximately 1% of the pediatric population and account for 2%–5% of all pediatric emergency department presentations [1]. The impact of seizures on kidney health remains largely unexplored. Although acute kidney injury (AKI) has been anecdotally reported in patients with status epilepticus, likely related to rhabdomyolysis-induced nephropathy, systematic data are lacking [3–5]. As shown in other common pediatric conditions [6–11], we hypothesized that AKI may occur in a clinically relevant proportion of children presenting with tonic–clonic seizures. Accordingly, we aimed to determine the prevalence of AKI and to identify factors associated with its development in children presenting with a first febrile or non-febrile tonic–clonic seizure. We decided to enroll only patients at their first febrile seizure to capture, as accurately as possible, the effect of a single seizure. This is because, in patients with recurrent seizures, the occurrence of AKI during some episodes may increase the risk of subsequent AKI [12]. Indeed, each AKI episode can reduce nephron mass and thereby increase susceptibility to further AKI under stressful conditions such as seizures [12, 13].

## Methods

We retrospectively collected data from all children consecutively presenting to the Pediatric Emergency Department of Sant’Anna e San Sebastiano Hospital, Caserta, Italy, for their first episode of acute tonic–clonic seizure between January 1, 2021, and July 31, 2025. The pediatric ward is located within a general hospital primarily devoted to adult care, where pediatricians manage a wide range of conditions before referring patients to regional tertiary pediatric centers when needed.

According to the International League Against Epilepsy definition, seizures were defined as a transient occurrence of signs and/or symptoms resulting from abnormal, excessive, or synchronous neuronal activity in the brain [14]. In this study, we included patients who presented with tonic–clonic seizures accompanied by impaired consciousness, either with fever (referred to in the manuscript as febrile seizures) or without fever (referred to as non-febrile seizures). Patients were included regardless of hospital admission, provided that serum creatinine measurement was available during emergency department evaluation. For patients who were subsequently hospitalized, further serum creatinine measurements were also collected when available.

Inclusion criteria were as follows: (i) age < 18 years; (ii) admission for a first tonic–clonic seizure episode, either febrile or non-febrile; and (iii) availability of serum creatinine measurements at admission or within the first 24 h. A first seizure was defined as the first seizure episode in a patient’s lifetime. Patients with a history of previous seizures or a known diagnosis of epilepsy were not included. Moreover, we excluded patients with underlying conditions that could potentially affect both seizure occurrence and kidney outcomes (such as perinatal asphyxia, syndromic conditions, or inherited metabolic diseases). We also excluded patients with previously known nephro-urological diseases and those with central nervous system infection.

**Fig. 1:**
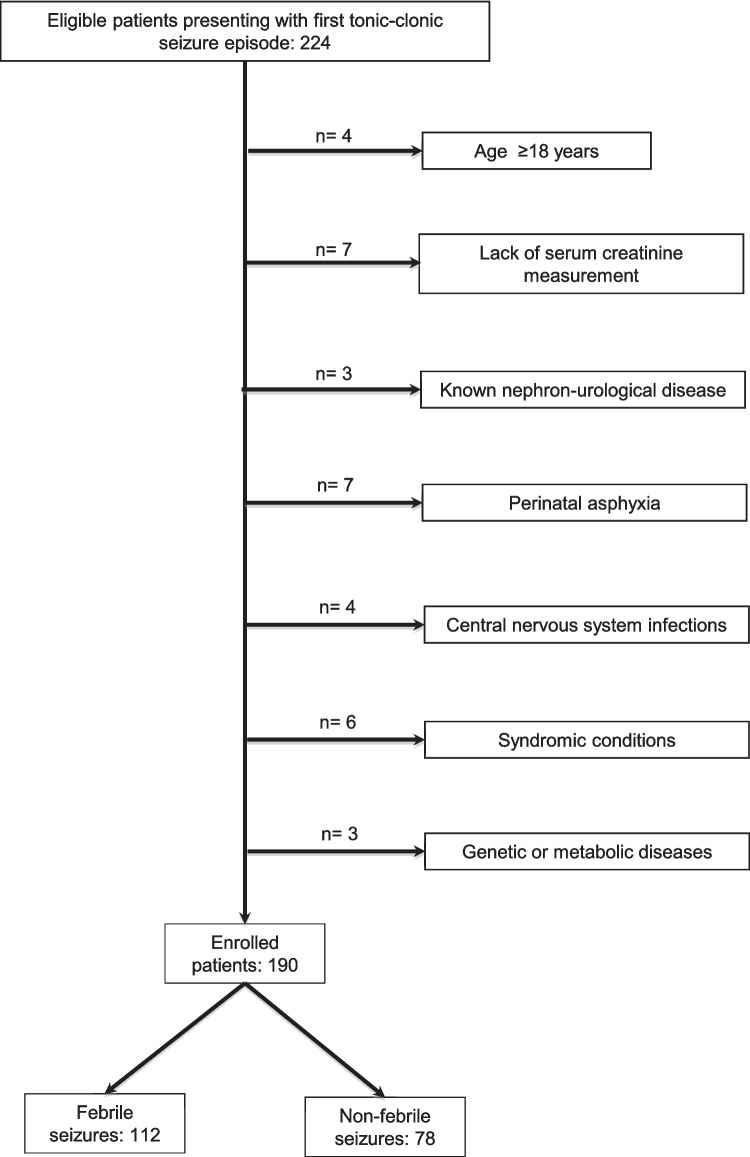
Consort diagram illustrating patient enrollment

The key variable was serum creatinine, as the study focuses on the development of AKI. Creatinine measurement was included in an emergency panel that comprised all the analyzed variables. The analytic cohort was restricted to patients with available serum creatinine measurements at admission or within 24 h, as required by the inclusion criteria. Therefore, a complete-case analysis was performed, and no missing data were present among the variables included in the final analyzed cohort. Patients without creatinine measurements *(n* = 7) were excluded from the primary analysis (Fig. 1) but were included in a sensitivity analysis assuming no AKI.

Moreover, as all patients presented with a first seizure, EEG was performed in every case in accordance with local hospital clinical practice, and this variable was also available for all patients. The study was conducted in accordance with the Declaration of Helsinki and was approved by the local Research Ethics Committee (approval no. 12770/2020). Written informed consent was obtained from the parents or legal guardians of all patients prior to any procedure. This consent was a general consent related to clinical procedures, given the patients’ conditions. A specific informed consent for study was waived by the Institutional Review Board due to the retrospective nature of the study and the use of anonymized data.

### Data collection

From the medical records, we extracted the variables listed in Table 1. All serum creatinine values measured during hospitalization were recorded. A second serum creatinine measurement was available for 97 patients who were hospitalized: in 59 patients after 48 h, in 25 after 72 h, in 10 after 96 h, and in 3 after 120 h.

**Table 1:** Clinical and laboratory characteristics of children evaluated at pediatric emergency department for febrile seizures with and without AKI

	AKI (no) No.= 98	AKI (yes) No.= 14	*p*
Age, yr, median (IQR)	1.9 (1.7)	2.2 (1.5)	0.71
Male gender, No. (%)	64 (65.3)	6 (42.9)	0.10
Birth weight, kg, median (IQR)	3.1 (0.6)	2.9 (1.1)	0.37
SGA, No. (%)	13 (13.3)	7 (50.0)	<0.001
Gestational age, weeks, median (IQR)	38 (2)	39 (2)	0.15
Preterm birth, No. (%)	11 (11.2)	1 (7.1)	0.64
Duration of fever before admission, hours, median (IQR)	2.0 (22.4)	9.0 (23.1)	0.20
Duration of seizures, min, median (IQR)	3.0 (3.0)	4.0 (3.3)	0.42
Complex seizures, No. (%)	35 (35.7)	4 (28.6)	0.77
Benzodiazepines utilization, No. (%)	35 (35.7)	1 (7.1)	0.04
Body temperature, peak at seizure, °C, median (IQR)	38.8 (0.5)	39.0 (1.2)	0.06
Paracetamol before admission, No. (%)	43 (43.9)	9 (64.3)	0.15
Ibuprofen before admission, No. (%)	18 (18.4)	7 (50.0)	0.008
Paracetamol+ibuprofen before admission, No. (%)	8 (8.2)	6 (42.9)	<0.001
Vomiting, No. (%)	12 (12.2)	1 (7.1)	0.99
Diarrhea, No (%)	15 (15.3)	2 (14.3)	0.99
Refill >2 seconds, No. (%)	1 (1.0)	0 (0)	0.99
Antibiotics administration, No. (%)	30 (30.6)	5 (35.7)	0.76
Need of intravenous rehydration, No. (%)	35 (35.7)	5 (35.7)	0.93
WBC, n/mcL, median (IQR)	12155 (9305)	15870 (11700)	0.08
Neutrophils, n/mcL, median (IQR)	8190 (7315)	7150 (7728)	0.82
Platelets, n/mcL, median (IQR)	267000 (121250)	297000 (143500)	0.30
Serum creatinine levels at admission, mg/dL, median (IQR)	0.30 (0.10)	0.45 (0.18)	<0.001
HC/BC ratio, median (IQR)	0.92 (0.33)	1.65 (0.41)	<0.001
Serum urea levels, mg/dL, mean (SDS)	11.5 ± 3.2	10.8 ± 3.2	0.41
Glycemia, mg/dL, median (IQR)	110 (34)	95 (34)	0.04
Hypoglicemia, No. (%)	0 (0)	1 (7.1)	0.12
Serum Na, mEq/L, mean (SDS)	134.5 ± 2.9	134.6 ± 3.6	0.71
Hyponatremia, No. (%)	18 (18.4)	2 (14.3)	0.99
Hypernatremia, No. (%)	0	0	N/A
Serum K, mEq/L, median (IQR)	4.1 (0.6)	4.3 (0.6)	0.46
Hypokalemia, No. (%)	3 (3.1)	0 (0)	0.99
Hyperkalemia, No. (%)	0	0	N/A
Serum Cl, mEq/L, mean (SDS)	101.8 ± 3.5	102 ± 4.0	0.57
Serum Ca, mg/dL, mean (SDS)	9.5 ± 0.6	9.4 ± 0.5	0.77
Hypocalcemia, No. (%)	3 (3.1)	0 (0)	0.99
Hypercalcemia, No. (%)	0	0	N/A
CPK, U/L, median (IQR)	110.5 (75)	84.5 (122)	0.11
Bicarbonates, mmol/L, mean (SDS)	22.6 ± 1.6	22.5 ± 3.2	0.94
Acidosis, No. (%)	5 (5.1)	1 (7.1)	0.56
CRP, mg/dL, median (IQR)	0.83 (2.12)	4.7 (10.0)	< 0.001
EEG abnormalities, No. (%)	3 (3.1)	3 (21.4)	0.03

For normal distributed variables means ± SDS are shown, while for non-parametric ones median and interquartile range are shown

*AKI* acute kidney injury; *Ca* calcium; *Cl* chloride; *CPK* creatine phosphokinase; *CRP* c-reactive protein; *EEG* electroencephalography; *HC/BC* highest-to-basal creatinine ratio; *IQR* interquartile range; *K* potassium; *Na* sodium; *SDS* standard deviation score; *SGA* small for gestational age; *WBC* white blood cells

### Case definition

The primary outcome was AKI, defined according to the Kidney Disease: Improving Global Outcomes (KDIGO) criteria based on serum creatinine [15]. Because baseline (premorbid) serum creatinine values were unavailable for all enrolled patients, baseline creatinine was estimated using previously validated back-calculation methods [16]. As previously reported, both height-dependent and height-independent approaches for estimating baseline serum creatinine perform comparably [16]. Accordingly, baseline serum creatinine was back-calculated using the Hoste age-based equation, assuming a baseline estimated glomerular filtration rate (eGFR) equal to the median age-specific normative eGFR values for children ≤ 2 years of age [17], and an eGFR of 120 mL/min/1.73 m^2^ for children older than 2 years [18]. This approach has also been validated in younger pediatric populations [10]. AKI was considered absent when all serum creatinine values remained < 1.5 times the estimated baseline value. Stage 1 AKI was defined by a creatinine increase of 1.5 to < 2 times baseline, stage 2 by an increase of 2 to < 3 times baseline, and stage 3 by an increase ≥ 3 times baseline [15].

The urine output component of the KDIGO criteria was not applied because urine output data were unavailable.

### Other definitions

Electroencephalography (EEG) abnormalities were defined as the presence of epileptiform graphoelements [19].

Fever was defined as an axillary body temperature > 38 °C at the time of seizure or during clinical evaluation in the emergency department and is referred to as the peak body temperature at seizure throughout the manuscript [20].

Hypoglycemia was defined as a serum glucose level < 50 mg/dL [21]. Hyponatremia and hypernatremia were defined as serum sodium levels < 135 mEq/L and > 145 mEq/L, respectively, whereas hypokalemia and hyperkalemia were defined as serum potassium levels < 3.5 mEq/L and > 5.5 mEq/L, respectively [22]. Acidosis was defined as a venous bicarbonate level < 22 mmol/L [23].

### Statistical analysis

Febrile and non-febrile seizures are distinct clinical entities, with different underlying mechanisms, triggers, and systemic inflammatory profiles, which could plausibly influence the pathophysiology of AKI. Therefore, we a priori decided to analyze patients with and without febrile seizures separately [24, 25].

A *p* value < 0.05 was considered statistically significant. Continuous variables were compared using the independent-sample *t* test for normally distributed data and the Mann–Whitney test for non-normally distributed data. The distribution of continuous variables was assessed using skewness and kurtosis, with values between − 2 and + 2 considered indicative of normal distribution.

Categorical variables were compared using the chi-square test or Fisher’s exact test, as appropriate.

Logistic regression models were applied to explore factors associated with AKI. Given the limited number of AKI events, a parsimonious approach was adopted to reduce the risk of overfitting, and only variables showing stronger statistical evidence of association were included in univariable and multiple logistic regression models. Therefore, variables showing a significant association with AKI (*p* < 0.05) in the comparison between patients with and without AKI (Table 1) were included in the univariate logistic regression analyses. Serum creatinine levels and the highest-to-basal creatinine (HC/BC) ratio were not included in the exploratory univariate analyses, as they were used to define the presence or absence of AKI. Univariate analyses were performed to identify candidate variables for inclusion in the multivariate models. Only variables with *p* < 0.05 at univariate analysis were entered into the exploratory multivariate analyses. In the febrile seizure subgroup, ibuprofen use was not included in the multivariate model because of high collinearity with combined ibuprofen plus paracetamol use and its weaker association with AKI. In multivariate analyses, only variables remaining significant after Bonferroni correction were considered statistically significant.

For these exploratory logistic regression analyses, internal validation using bootstrap resampling (1000 iterations) and a leave-one-variable-out sensitivity analysis were also performed to confirm the observed associations.

The association between C-reactive protein (CRP) and peak body temperature at seizure was analyzed using linear regression.

As a secondary analysis, an exploratory multivariable logistic regression model was performed in the overall cohort including seizure type as a covariate. Given the limited number of events, a parsimonious approach was adopted, and only CRP—identified as the only variable consistently associated with AKI across subgroup analyses—was included in the model.

All statistical analyses were performed using SPSS version 25 for Mac.

## Results

### General characteristics of overall population

A total of 224 patients were eligible for inclusion. After excluding those who did not meet the inclusion criteria or met any of the exclusion criteria, 190 children were enrolled, with a mean age of 4.2 ± 3.7 years (Fig. 1). Of these, 112 (58.9%) presented with febrile seizures (Fig. 1). A total of 97 patients were hospitalized following evaluation in the Pediatric Emergency Department. Median PCR levels were similar between hospitalized and non-hospitalized patients (0.65, IQR 2.30 vs 0.55, IQR 1.33; *p* = 0.18). AKI prevalence was also similar between the two groups (52.2% vs 47.8%; *p* = 0.91). The general characteristics of patients with febrile and non-febrile seizures are shown in Supplementary Table 1. According to the definition, none of the patients with non-febrile seizures presented with fever.

Patients with febrile seizures were younger, had a similar prevalence of male sex, a higher use of paracetamol, ibuprofen, or their combination, a higher prevalence of diarrhea and antibiotic administration, and a lower prevalence of EEG abnormalities compared with patients with non-febrile seizures. Moreover, patients with febrile seizures had higher levels of white blood cells, neutrophils, glycemia, and CRP, and lower levels of platelets, sodium, chloride, and bicarbonate compared with patients with non-febrile seizures (Supplementary Table 1).

Among patients with febrile seizures, a statistically significant linear relationship was observed between CRP levels and peak body temperature at seizure (*r*^2^ = 0.043; *F*-ratio = 4.9; *p* = 0.03).

Among the 112 patients with febrile seizures, only six exhibited concomitant EEG abnormalities. The underlying cause of fever was unknown in 51 patients (45.5%), although all showed symptoms suggestive of a viral infection with an unidentified pathogen. Two patients (1.8%) developed fever following the Measles–Mumps–Rubella vaccine, 41 (36.6%) had infections caused by identified viruses, 4 (3.6%) had febrile urinary tract infections, 5 (4.5%) had bacterial lower respiratory tract infections, and 9 (8.0%) had bacterial upper urinary tract infections. The prevalence of AKI was similar in patients with an unidentified versus an identified cause of fever (9.6% vs. 15.0%, *p* = 0.57).

Among the 78 patients with non-febrile seizures, 39 (56.5%) exhibited EEG abnormalities, 3 (3.8%) had seizures secondary to head trauma, 3 (3.8%) had seizures due to hypoglycemia, and 33 (42.3%) had seizures of unidentified cause. The prevalence of AKI was similar in patients with an unidentified versus an identified cause of non-febrile seizures (11.1% vs. 12.1%, *p* = 0.99).

### AKI characteristics

**Fig. 2:**
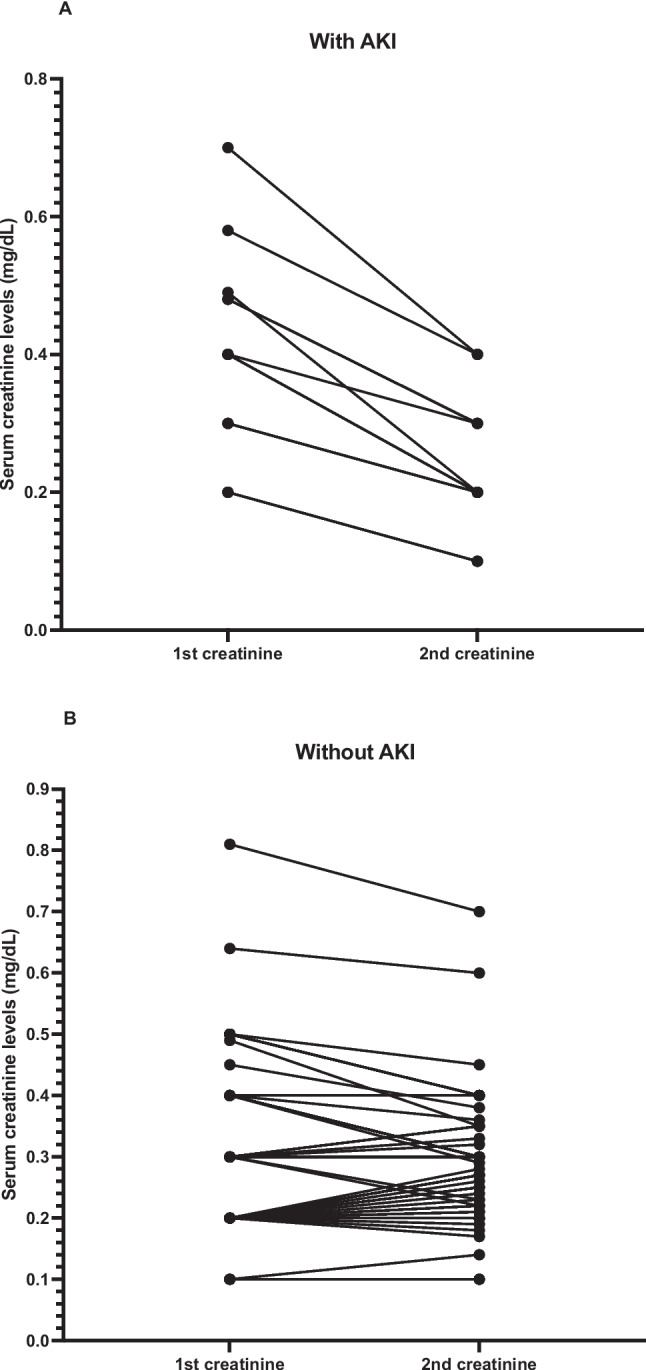
Creatinine trajectories in 97 patients with a second measurement, stratified by AKI development. **A** Patients with AKI; **B** patients without AKI

AKI occurred in 23 of 190 patients (12.1%). A sensitivity analysis including the 7 patients without serum creatinine measurement within 24 h, assuming no AKI in this group, yielded a similar AKI prevalence (11.7% vs. 12.1% in the primary analysis; *p* = 0.89). Trajectories of creatinine levels for the 97 patients with a second creatinine measurement, stratified by AKI development, are shown in Fig. 2A and B. The delta in serum creatinine (first minus second value) was 0.17 ± 0.07 in patients with AKI and 0.02 ± 0.06 in those without AKI (*p* < 0.001).

AKI was observed in 14 of 112 patients (12.5%) with febrile seizures and in 9 of 78 patients (11.5%) with non-febrile seizures (*p* = 0.84). None of the patients required hemodialysis. Among patients with AKI and febrile seizures, 10 reached AKI stage 1 and 4 reached stage 2. Among patients with AKI and non-febrile seizures, 7 reached stage 1 and 2 reached stage 2. In all cases, the maximum AKI stage was already present at hospital admission.

### Factors associated with AKI development

#### Patients with febrile seizures

Among patients with febrile seizures, those who developed AKI showed a higher prevalence of being born small for gestational age (SGA), a higher rate of prior administration of ibuprofen and combined paracetamol plus ibuprofen before admission, EEG abnormalities, and higher CRP levels compared with patients without AKI.

In univariate logistic regression analysis, being born SGA, administration of ibuprofen or ibuprofen plus paracetamol, CRP levels, and EEG abnormalities were significantly associated with the development of AKI (Table 2). After Bonferroni correction, only being born SGA, administration of ibuprofen plus paracetamol, and CRP abnormalities remained significantly associated with AKI (Table 2). In addition, we performed an internal validation using bootstrap resampling (1000 iterations), which confirmed statistically significant associations between AKI and SGA (*p* = 0.005), combined paracetamol plus ibuprofen use (*p* = 0.001), CRP (*p* = 0.003), and EEG abnormalities (*p* = 0.004). A leave-one-variable-out sensitivity analysis further supported the robustness of the findings, as the association between CRP and AKI remained statistically significant with similar odds ratios across all models.

**Table 2:** Univariate and multiple logistic regression for factors associated with AKI in children with febrile seizures

	Univariate			Multiple		
	OR	95%CI	*p*	OR	95%CI	p*
SGA	4.9	1.5–16.4	0.01	7.2	1.5–35.1	0.01
Ibuprofen	4.4	1.4–14.3	0.01	–	–	–
Paracetamol + Ibuprofen	6.2	1.7–23.3	0.006	11.2	2.1–61.3	0.005
CRP	1.2	1.03–1.3	0.01	1.2	1.1–1.4	0.005
EEG abnormalities	8.7	1.6–48.1	0.01	14.3	1.3–158.3	0.03

*After Bonferroni correction, the *p*-value threshold was set at 0.012

*AKI* acute kidney injury; *CI* confidence interval; *CRP* c-reactive protein; *EEG* electroencephalography; *OR* odds ratio; SGA, small for gestational age

#### Patients with non-febrile seizures

Among patients with non-febrile seizures, those who developed AKI had a longer seizure duration and higher CRP levels compared with patients who did not develop AKI (Table 3). Both factors were significantly associated with AKI development in univariate analysis; however, after Bonferroni correction in multivariable logistic regression, only CRP levels remained significantly associated with AKI (Table 4). Internal validation using bootstrap resampling (1000 iterations) confirmed a significant association between AKI and CRP (*p* = 0.01), and leave-one-variable-out sensitivity analysis yielded consistent results.

**Table 3:** Clinical and laboratory characteristics of children evaluated at pediatric emergency department for non-febrile seizures with and without AKI

	AKI (no) No.= 69	AKI (yes) No.= 9	*p*
Age, yr, median (IQR)	7.0 (7.0)	7.9 (4.6)	0.45
Male gender, No. (%)	47 (68.1)	6 (66.7)	0.93
Birth weight, kg, median (IQR)	3.2 (0.56)	3.2 (1.1)	0.59
SGA, No. (%)	7 (10.1)	1 (11.1)	0.99
Gestational age, weeks, median (IQR)	38.0 (2.0)	39.0 (3.0)	0.88
Preterm birth, No. (%)	7 (10.1)	1 (11.1)	0.99
Duration of seizures, min, median (IQR)	4.0 (8.0)	10.0 (8.0)	0.005
Benzodiazepines utilization, No. (%)	17 (24.6)	3 (33.3)	0.69
Vomiting, No. (%)	10 (14.5)	3 (33.3)	0.17
Diarrhea, No (%)	4 (5.8)	0 (0)	0.99
Refill >2 seconds, No. (%)	1 (1.4)	0 (0)	0.87
Antibiotics administration, No. (%)	4 (5.8)	1 (11.1)	0.54
Need of intravenous rehydration, No. (%)	24 (34.8)	5 (55.6)	0.46
WBC, n/mcL, median (IQR)	9005 (3643)	9780 (5745)	0.41
Neutrophils, n/mcL, median (IQR)	4175 (3136)	6300 (4980)	0.28
Platelets, n/mcL, median (IQR)	301000 (123750)	325000 (120500)	0.98
Serum creatinine levels at admission, mg/dL, median (IQR)	0.30 (0.25)	0.66 (0.13)	< 0.001
HC/BC ratio, median (IQR)	0.84 (0.42)	1.60 (0.44)	< 0.001
Serum urea levels, mg/dL, mean (SDS)	11.6 (3.3)	12.2 (2.3)	0.61
Glycemia, mg/dL, median (IQR)	92.0 (26.0)	98 (46.0)	0.47
Serum Na, mEq/L, mean (SDS)	137.4 (2.6)	138.6 (3.2)	0.22
Serum K, mEq/L, median (IQR)	4.2 (0.8)	4.1 (0.8)	0.45
Serum Cl, mEq/L, mean (SDS)	103.8 (3.0)	104.9 (3.9)	0.32
Serum Ca, mg/dL, mean (SDS)	9.6 (0.49)	9.8 (0.35)	0.31
CPK, U/L, median (IQR)	115 (103)	141 (129)	0.91
Bicarbonates, mmol/L, mean (SDS)	24.3 (2.9)	26.0 (2.2)	0.19
CRP, mg/dL, median (IQR)	0.06 (0.36)	1.4 (0.95)	< 0.001
EEG abnormalities, No. (%)	39 (56.5)	3 (33.3)	0.28

For normal distributed variables means ± SDS are shown, while for non-parametric ones median and interquartile range are shown

*AKI* acute kidney injury; *Ca* calcium; *Cl* chloride; *CPK* creatine phosphokinase; *CRP* c-reactive protein; *EEG* electroencephalography; *HC/BC* highest-to-basal creatinine ratio; *IQR* interquartile range; *K* potassium; *Na* sodium; *SDS* standard deviation score; *SGA* small for gestational age; *WBC* white blood cells

**Table 4:** Univariate and multiple logistic regression for factors associated with AKI in children with non-febrile seizures

	Univariate			Multiple		
	OR	95%CI	*p*	OR	95%CI	p*
Duration of seizures	1.1	1.05–1.1	0.039	1.03	0.91–1.17	0.63
CRP	7.9	2.30–26.7	<0.001	6.9	1.9–25.1	0.003

*After Bonferroni correction, the *p*-value threshold was set at 0.025

*AKI* acute kidney injury; *CI* confidence interval; *CRP* c-reactive protein; OR, odds ratio

#### Both the febrile and non-febrile seizure patient groups

In an overall multivariable model including seizure type as a covariate and CRP as predictor, CRP (OR = 1.29; 95%CI: 1.11–1.48; *p* < 0.001) remained significantly associated with AKI, whereas seizure type was not independently associated (OR = 2.08; 95%CI 0.72–6.1; *p* = 0.18).

## Discussion

Our study analyzes the prevalence of and factors associated with AKI in children presenting with a first tonic–clonic seizure. AKI is often a neglected diagnosis in children [26] because it is self-resolving in most cases; however, it can complicate several common pediatric conditions [6–10, 13, 27, 28]. Reported prevalence rates vary widely, ranging from 7.4% in acute appendicitis [6] to 65.2% in diabetic ketoacidosis [11], with intermediate rates of 14.6% in febrile urinary tract infection [10], 17.6% in acute bronchiolitis [8], 20.4% in community-acquired pneumonia [9], and 24.6% in acute gastroenteritis [7]. We found that approximately 12% of children with tonic–clonic seizures, both febrile and non-febrile, developed AKI, with a similar prevalence in the two groups. The prevalence of AKI was also comparable between patients with and without an identified cause of non-febrile seizures, as well as between those with and without an identified cause of fever among patients with febrile seizures. An underlying cause was not identified in approximately 42% of patients with non-febrile seizures, which is consistent with reports in the literature indicating that up to 50% of such cases remain unexplained after the initial clinical evaluation [29–31].

To date, no studies have evaluated the prevalence of AKI in either pediatric or adult cohorts with tonic–clonic seizures. Therefore, direct comparisons with existing literature are not possible. Anecdotal reports have documented AKI in patients with persistent non-febrile tonic–clonic seizures, with the underlying pathophysiological mechanism thought to potentially involve seizure-induced skeletal muscle injury, possibly leading to rhabdomyolysis and subsequent renal dysfunction [3–5].

In line with these findings, in the subgroup of patients with non-febrile tonic–clonic seizures, exploratory univariate logistic regression analysis showed that seizure duration was associated with AKI development, with the odds increasing by approximately 1.1-fold for each additional minute of seizure activity. However, in the exploratory multivariable logistic regression model, CRP levels exhibited a stronger association with AKI and remained the only variable independently associated with AKI.

This appears to not support the hypothesis derived from anecdotal cases in the literature [3–5], namely that rhabdomyolysis is involved in the pathophysiology of AKI in this setting, and suggests that the underlying mechanisms are in fact different. However, we only had data from a single creatine phosphokinase (CPK) measurement, with no information on CPK trends or other direct markers (e.g., aldolase, lactate dehydrogenase, and aspartate aminotransferase).

According to previous literature [32, 33], although we observed an association between a nonspecific marker such as CRP and AKI, our finding may be consistent with the hypothesis that seizures are associated with neuroinflammatory processes extending beyond the central nervous system, potentially contributing to systemic inflammation. In turn, systemic inflammation may be associated with AKI, as reported in other inflammatory conditions [6, 9, 10, 28].

Moreover, in the analysis restricted to febrile seizures, multivariable logistic regression confirmed that CRP levels remained significantly associated with AKI, further supporting a potential role of inflammation in the development of AKI. In addition, multivariable analysis indicated that being born SGA and the use of combined paracetamol and ibuprofen were also independently associated with AKI. The association with SGA is of interest from a pathophysiological perspective, as SGA status has been associated with reduced nephron endowment and may therefore confer increased susceptibility to AKI under stressful conditions [34, 35]. The association with combined paracetamol and ibuprofen use may have relevant clinical implications. While causality cannot be inferred, these findings may suggest caution regarding the concomitant use of these medications, potentially favoring paracetamol alone in patients at increased risk of febrile seizures. Ibuprofen, in fact, has been shown to reduce renal blood flow, particularly in settings that predispose children to dehydration or hypovolemia, which may contribute to an increased risk of AKI [36, 37].

In this cohort, AKI appeared to be mild; indeed, no difference in AKI prevalence was observed between patients who were hospitalized and those who were not after evaluation in the Pediatric Emergency Department. Therefore, in this setting, AKI may have limited relevance in the acute phase, as it is mild and self-resolving, but it remains clinically important in the long term.

From a practical standpoint, indeed, our findings may be clinically relevant, as suspecting and diagnosing AKI in children presenting with seizures could help identify those at higher risk of developing chronic kidney disease (CKD) [13, 38]. Even a single episode of mild AKI has been shown to approximately double the risk of CKD, with the risk increasing further in more severe cases [38]. Moreover, because seizures often recur [39, 40], awareness of AKI risk factors in these patients may help prevent recurrent AKI episodes, which is particularly important given that recurrent AKI is associated with an increased risk of CKD [41].

This study has several limitations. The retrospective design of the study and the single-center recruitment represent an inherent limitation. The incidence of AKI and its associated factors in children with seizures may vary considerably across geographic regions, centers, and patient demographics; however, our study design did not allow us to explore this potential variability. The lack of detailed long-term neurological follow-up also precluded the assessment of potential associations between AKI and specific epilepsy syndromes. However, among children with non-febrile seizures, the prevalence of EEG abnormalities was similar in those with and without AKI, and by restricting the analysis to first episodes of tonic–clonic seizures we aimed to reduce clinical heterogeneity and focus on a presentation commonly encountered in emergency settings.

The small sample size of patients with AKI could have led to model overfitting. Nevertheless, the bootstrap resampling, the sensitivity analysis, and the biological plausibility of the associations support the robustness of our findings. In addition, the variable selection strategy, based on a restrictive statistical approach, may have led to the exclusion of predictors with weaker but potentially clinically relevant associations and did not allow the assessment of possible interactions between variables. We acknowledge, however, that the limited number of events precludes definitive conclusions and that several estimates in the multivariable analyses show wide confidence intervals, indicating limited precision; therefore, these results should be considered hypothesis-generating and require confirmation in larger, prospective, multicenter studies. While analyzing patients with febrile and non-febrile seizures together could have increased the power of the study, we decided a priori to analyze the two groups separately. This approach was chosen because the pathophysiological mechanisms and underlying causes differ significantly between febrile and non-febrile seizures. The absence of measured baseline serum creatinine values represents an additional limitation; however, baseline estimation methods are widely validated in pediatric populations and otherwise healthy children do not routinely undergo blood testing. The use of estimated baseline creatinine may have introduced some misclassification in AKI diagnosis. Since baseline values were unavailable, direct verification of AKI classification was not possible, and sensitivity analyses using stricter AKI definitions were not feasible due to the very low number of cases. Nonetheless, this limitation is more likely to have attenuated the strength of the observed associations rather than produced false-positive results.

Although elevated CRP levels were independently associated with AKI, the observational nature of the study precludes causal inference, as CRP may reflect pre-existing or seizure-related systemic inflammation or overall disease severity. Nevertheless, the consistency of this association across seizure subgroups supports a potential role of inflammation in AKI susceptibility. Moreover, we did not systematically collect data on patients’ fluid and food intake prior to admission; however, we used surrogate markers—capillary refill time and the need for intravenous rehydration—which were available in the clinical charts.

Finally, the association between combined paracetamol and ibuprofen use and AKI may be influenced by confounding by indication; therefore, causality cannot be inferred. However, given the known effects of non-steroidal anti-inflammatory drugs on renal perfusion, particularly in dehydrated states, these findings support a cautious approach to antipyretic use in children at risk of AKI.

## Conclusions

In conclusion, AKI occurs in approximately 12% of children presenting with a first tonic–clonic seizure, whether febrile or non-febrile. Being born SGA, elevated CRP levels, and the combined use of paracetamol and ibuprofen were associated with AKI in children with febrile seizures, whereas elevated CRP levels were associated with AKI in those with non-febrile seizures. However, given the limited number of events, these findings should be interpreted as exploratory.

## Supplementary Information

Below is the link to the electronic supplementary material.

ESM 1 (DOC 72.5 KB)

## Data Availability

The datasets used and/or analysed during the current study are available from the corresponding author on reasonable request.

## Abbreviations

AKI: Acute kidney injury
CRP: C-reactive protein
EEG: Electroencephalography
eGFR: Estimated glomerular filtration rate
HC/BC: Highest-to-basal creatinine
KDIGO: Kidney Disease: Improving Global Outcomes
SGA: Small for gestational age

## Code availability

N/A.

## References

[1] Bergamo S, Parata F, Nosadini M et al (2015) Children with convulsive epileptic seizures presenting to Padua pediatric emergency department: the first retrospective population-based descriptive study in an Italian health district. J Child Neurol 30:289–295. 10.1177/0883073814538670 25008906 10.1177/0883073814538670

[2] França UL, McManus ML (2020) Assessment of acute hospital use and transfers for management of pediatric seizures. JAMA Netw Open 3:E203148. 10.1001/JAMANETWORKOPEN.2020.3148 32315068 10.1001/jamanetworkopen.2020.3148 PMC7175083

[3] Tomomitsu Y, Asakawa S, Arai S et al (2022) A patient with acute kidney injury associated with massive proteinuria and acute hyperuricemia after epileptic seizures. Intern Med 61:3401–3408. 10.2169/INTERNALMEDICINE.8808-21 35466163 10.2169/internalmedicine.8808-21 PMC9751716

[4] Wang L, Hong S, Huang H, Yang M (2018) Rhabdomyolysis following status epilepticus with hyperuricemia. Medicine (United States) 97:e11281. 10.1097/MD.0000000000011281 10.1097/MD.0000000000011281 PMC6039628 29953009

[5] Makki N, Hajj G, Schmidt GA (2013) Seizure-induced acute urate nephropathy: case report and review. Chest 144:666–669. 10.1378/CHEST.12-2129 23918111 10.1378/chest.12-2129

[6] Marzuillo P, Coppola C, Caiazzo R et al (2022) Acute kidney injury in children with acute appendicitis. Children Basel 9:620. 10.3390/CHILDREN9050620 35626797 10.3390/children9050620 PMC9139852

[7] Marzuillo P, Baldascino M, Guarino S et al (2021) Acute kidney injury in children hospitalized for acute gastroenteritis: prevalence and risk factors. Pediatr Nephrol 36:1627–1635. 10.1007/s00467-020-04834-7 33411074 10.1007/s00467-020-04834-7 PMC8084840

[8] Marzuillo P, Di Sessa A, Golino R et al (2023) Acute kidney injury in infants hospitalized for viral bronchiolitis. Eur J Pediatr 182:3569–3576. 10.1007/S00431-023-05029-6 37222853 10.1007/s00431-023-05029-6 PMC10205560

[9] Marzuillo P, Pezzella V, Guarino S et al (2021) Acute kidney injury in children hospitalized for community acquired pneumonia. Pediatr Nephrol 36:2883–2890. 10.1007/s00467-021-05022-x 33745060 10.1007/s00467-021-05022-x PMC8370910

[10] Marzuillo P, Guarino S, Alfiero S et al (2024) Acute kidney injury in children hospitalised for febrile urinary tract infection. Acta Paediatr. 10.1111/APA.17247 10.1111/apa.17247 38641985

[11] Marzuillo P, Iafusco D, Zanfardino A et al (2021) Acute kidney injury and renal tubular damage in children with type 1 diabetes mellitus onset. J Clin Endocrinol Metab 106:e2720–e2737. 10.1210/clinem/dgab090 33595665 10.1210/clinem/dgab090

[12] Liu KD, Yang J, Tan TC et al (2019) Risk factors for recurrent acute kidney injury in a large population-based cohort. Am J Kidney Dis 73:163–173. 10.1053/J.AJKD.2018.08.008 30482577 10.1053/j.ajkd.2018.08.008 PMC6647831

[13] Rivetti G, Gizzone P, Petrone D et al (2024) Acute kidney injury in children: a focus for the general pediatrician. Children Basel 11:1004. 10.3390/CHILDREN11081004 39201939 10.3390/children11081004 PMC11352805

[14] Fisher RS, Cross JH, French JA et al (2017) Operational classification of seizure types by the International League Against Epilepsy: position paper of the ILAE Commission for Classification and Terminology. Epilepsia 58:522–530. 10.1111/EPI.13670 28276060 10.1111/epi.13670

[15] Kellum JA, Lameire N, Aspelin P, et al (2012) Kidney disease: improving global outcomes (KDIGO) acute kidney injury work group. KDIGO clinical practice guideline for acute kidney injury. Kidney Int Suppl (2011) 2:1–138. 10.1038/kisup.2012.1

[16] Hessey E, Ali R, Dorais M et al (2017) Evaluation of height-dependent and height-independent methods of estimating baseline serum creatinine in critically ill children. Pediatr Nephrol 32:1953–1962. 10.1007/s00467-017-3670-z 28523356 10.1007/s00467-017-3670-z

[17] Piepsz A, Tondeur M, Ham H (2006) Revisiting normal 51Cr-ethylenediaminetetraacetic acid clearance values in children. Eur J Nucl Med Mol Imaging 33:1477–1482. 10.1007/s00259-006-0179-2 16865393 10.1007/s00259-006-0179-2

[18] Schwartz GJ, Work DF (2009) Measurement and estimation of GFR in children and adolescents. Clin J Am Soc Nephrol 4:1832–1843. 10.2215/CJN.01640309 19820136 10.2215/CJN.01640309

[19] Alcala-Zermeno JL, Katyal R, Frauscher B et al (2025) Seminar in epileptology: normal awake and sleep patterns, interictal abnormalities, and ictal patterns on scalp EEG. Epileptic Disord 27:803–866. 10.1002/EPD2.70071 40782030 10.1002/epd2.70071 PMC12574496

[20] Barbi E, Marzuillo P, Neri E et al (2017) Fever in children: pearls and pitfalls. Children 4:81. 10.3390/children4090081 28862659 10.3390/children4090081 PMC5615271

[21] Quarta A, Iannucci D, Guarino M et al (2023) Hypoglycemia in children: major endocrine-metabolic causes and novel therapeutic perspectives. Nutrients 15:3544. 10.3390/NU15163544 37630734 10.3390/nu15163544 PMC10459037

[22] Marzuillo P, Guarino S, Annicchiarico Petruzzelli L et al (2024) Prevalence of and factors associated with Na + /K + imbalances in a population of children hospitalized with febrile urinary tract infection. Eur J Pediatr 183:5223–5232. 10.1007/S00431-024-05784-0 39356305 10.1007/s00431-024-05784-0 PMC11527937

[23] Guarino S, Iafusco D, Di Sessa A et al (2025) Acidosis at diagnosis of type 1 diabetes mellitus: relation with kidney function. Pediatr Diabetes 2025:3188571. 10.1155/PEDI/3188571 41293549 10.1155/pedi/3188571 PMC12643667

[24] Paige AL, Cavanna AE (2023) Generalized tonic-clonic seizure. Neuroimaging of Consciousness 81–97. 10.1007/978-3-642-37580-4_6

[25] Tiwari A, Meshram RJ, Singh RK (2022) Febrile seizures in children: a review. Cureus 14:e31509. 10.7759/CUREUS.31509 36540525 10.7759/cureus.31509 PMC9754740

[26] Rivetti G, Marzuillo P (2023) Community-acquired acute kidney injury in hospitalized children: do not miss the diagnosis! Indian Pediatr 60:433–434 37293905

[27] Rivetti G, Hursh BE, Miraglia del Giudice E, Marzuillo P (2023) Acute and chronic kidney complications in children with type 1 diabetes mellitus. Pediatr Nephrol 38:1449–1458. 10.1007/s00467-022-05689-w 35896816 10.1007/s00467-022-05689-w PMC10060299

[28] Gicchino MF, Arenella M, De Simone C et al (2026) Prevalence and risk factors for acute kidney injury at the diagnosis of juvenile idiopathic arthritis in children and its long-term implications for kidney health. Pediatr Nephrol. 10.1007/S00467-026-07222-9 41774201 10.1007/s00467-026-07222-9 PMC13197239

[29] Ochoa-Gómez L, López-Pisón J, Lapresta Moros C et al (2017) A study of epilepsy according to the age at onset and monitored for 3 years in a regional reference paediatric neurology unit. An Pediatr (Barc) 86:11–19. 10.1016/J.ANPEDI.2016.05.002 27291698 10.1016/j.anpedi.2016.05.002

[30] Araştirma Ö, Tütüncü Toker R, Uludağ B (2023) The Journal of Current Pediatrics Güncel Pediatri. J Curr Pediatr 21:195–201. 10.4274/jcp.2023.74508

[31] DrM A, DrPP S, DrS A et al (2025) Clinical profile, etiology and outcome of afebrile seizures in children. International Journal of Medical and Pharmaceutical Research 6:869–874. 10.5281/ZENODO.17347010

[32] Zhong R, Chen Q, Li M et al (2019) Elevated blood C-reactive protein levels in patients with epilepsy: a systematic review and meta-analysis. Front Neurol 10:974. 10.3389/FNEUR.2019.00974 31620066 10.3389/fneur.2019.00974 PMC6759543

[33] Madžar D, Reindl C, Mrochen A et al (2021) Value of initial C-reactive protein levels in status epilepticus outcome prediction. Epilepsia 62:e48–e52. 10.1111/EPI.16842 33609292 10.1111/epi.16842

[34] Schreuder MF (2012) Safety in glomerular numbers. Pediatr Nephrol 27:1881–1887 22532329 10.1007/s00467-012-2169-x PMC3422453

[35] Luyckx VA, Brenner BM (2010) The clinical importance of nephron mass. J Am Soc Nephrol 21:898–910. 10.1681/ASN.2009121248 20150537 10.1681/ASN.2009121248

[36] Balestracci A, Ezquer M, Elmo ME et al (2015) Ibuprofen-associated acute kidney injury in dehydrated children with acute gastroenteritis. Pediatr Nephrol 30:1873–1878. 10.1007/S00467-015-3105-7 25895445 10.1007/s00467-015-3105-7

[37] Misurac JM, Grinsell MM, Narus JAH et al (2023) NSAID-associated acute kidney injury in hospitalized children - a prospective Pediatric Nephrology Research Consortium study. Pediatr Nephrol 38:3109–3116. 10.1007/S00467-023-05916-Y 36943469 10.1007/s00467-023-05916-y

[38] Coca SG, Singanamala S, Parikh CR (2012) Chronic kidney disease after acute kidney injury: a systematic review and meta-analysis. Kidney Int 81:442–448. 10.1038/ki.2011.379 22113526 10.1038/ki.2011.379 PMC3788581

[39] Maia C, Moreira AR, Lopes T, Martins C (2017) Risk of recurrence after a first unprovoked seizure in children. Jornal de Pediatria (English Edition) 93:281–286. 10.1016/J.JPED.2016.07.001 10.1016/j.jped.2016.07.001 27686587

[40] Chejety RR, Tejashwini K, Kumar D et al (2024) A clinical study on risk factors of febrile seizure recurrence among children. Eur J Cardiovasc Med 14:1127–1133. 10.5083/EJCM/2024

[41] Rodríguez E, Arias-Cabrales C, Bermejo S et al (2018) Impact of recurrent acute kidney injury on patient outcomes. Kidney Blood Press Res 43:34–44. 10.1159/000486744 29393217 10.1159/000486744

