# Supplementary material for: Prevalence and factors associated with acute kidney injury in children presenting to the emergency department with a first tonic–clonic seizure: an exploratory study
Source: Eur J Pediatr. 2026 May 22;185(6):429. doi: 10.1007/s00431-026-07079-y (PMC13197242; doi:10.1007/s00431-026-07079-y)
Supplement: Supplementary file 1 — (DOC 72.5 KB) [file 431_2026_7079_MOESM1_ESM.doc]

|  | **Non-febrile seizures**  **No.= 78** | **Febrile seizures**  **No.= 112** | **p** |
| --- | --- | --- | --- |
| **Age, yr, median (IQR)*^&^** | 7.0 (6.8) | 1.9 (1.7) | <0.001 |
| **Male gender, No. (%)** | 53 (67.9) | 70 (62.5) | 0.44 |
| **Birth weight, kg, median (IQR)** | 3.2 (0.58) | 3.1 (0.66) | 0.31 |
| **Small for gestational age, No. (%)** | 8 (10.3) | 20 (17.9) | 0.15 |
| **Gestational age, weeks, median (IQR)** | 38.0 (2.0) | 38.0 (2.0) | 0.63 |
| **Preterm birth, No. (%)** | 8 (10.3) | 12 (10.7) | 0.92 |
| **Duration of fever before admission, hours, median (IQR)** | N/A | 3.0 (23.1) | N/A |
| **Duration of seizures, min, median (IQR)** | 5.0 (8.0) | 3.0 (3.0) | 0.21 |
| **Complex seizures, No. (%)** | N/A | 39 (34.8) | N/A |
| **Benzodiazepines utilization, No. (%)** | 20 (25.6) | 36 (32.1) | 0.33 |
| **Fever, No. (%)** | 0 (0) | 112 (100) | <0.001 |
| **Paracetamol before admission, No. (%)** | 1 (1.3) | 52 (46.4) | <0.001 |
| **Ibuprofen before admission, No. (%)** | 1 (1.3) | 25 (22.3) | <0.001 |
| **Paracetamol+ibuprofen before admission, No. (%)** | 1 (1.3) | 14 (12.5) | 0.005 |
| **Vomiting, No. (%)** | 13 (16.7) | 13 (11.6) | 0.32 |
| **Diarrhea, No. (%)** | 4 (5.1) | 17 (15.2) | 0.03 |
| **Refill >2 seconds, No. (%)** | 1 (1.3) | 1 (0.9) | 0.47 |
| **Antibiotics administration, No. (%)** | 5 (6.4) | 35 (31.3) | <0.001 |
| **Need of intravenous rehydration, No. (%)** | 29 (37.2) | 40 (35.7) | 0.94 |
| **WBC, n/mcL, median (IQR)^&^** | 9430 (3710) | 12925 (8988) | 0.001 |
| **Neutrophils, n/mcL, median (IQR)^&^** | 4280 (3693) | 8790 (7364) | <0.001 |
| **Platelets, n/mcL, median (IQR)^&^** | 302000 (122500) | 275500 (121500) | 0.03 |
| **AKI, No. (%)** | 9 (11.5) | 14 (12.5) | 0.84 |
| **HC/BC ratio, median (IQR)** | 0.87 (0.44) | 0.92 (0.45) | 0.78 |
| **Serum urea levels, mg/dL, mean (SDS)** | 11.7 (3.2) | 11.4 (3.2) | 0.57 |
| **Glycemia, mg/dL, median (IQR)^&^** | 92.5 (26.0) | 108 (31.0) | <0.001 |
| **Hypoglicemia, No. (%)** | 2 (2.6) | 1 (0.89) | 0.57 |
| **Serum Na, mEq/L, mean (SDS)^&^** | 137.5 (2.7) | 134.5 (2.9) | <0.001 |
| **Hyponatremia, No. (%)** | 14 (17.9) | 20 (17.8) | 0.99 |
| **Hypernatremia, No. (%)** | 0 | 0 | N/A |
| **Serum K, mEq/L, median (IQR)** | 4.2 (0.7) | 4.1 (0.6) | 0.63 |
| **Hypokalemia, No. (%)** | 2 (2.6) | 3 (2.7) | 0.99 |
| **Hyperkalemia, No. (%)** | 0 | 0 | N/A |
| **Serum Cl, mEq/L, mean (SDS)^&^** | 103.9 (3.1) | 101.9 (3.6) | <0.001 |
| **Serum Ca, mg/dL, mean (SDS)** | 9.6 (0.48) | 9.5 (0.57) | 0.10 |
| **Hypocalcemia, No. (%)** | 0 (0) | 3 (2.7) | 0.27 |
| **Hypercalcemia, No. (%)** | 0 | 0 | N/A |
| **CPK, U/L, median (IQR)** | 116.5 (103.0) | 108.5 (79) | 0.83 |
| **Bicarbonates, mmol/L, mean (SDS)^&^** | 24.7 (2.9) | 22.6 (1.90) | 0.01 |
| **Acidosis, No. (%)** | 3 (3.8) | 6 (5.4) | 0.74 |
| **CRP, mg/dL, median (IQR)^&^** | 0.11 (0.56) | 1.0 (2.44) | <0.001 |
| **EEG abnormalities, No. (%)** | 42 (53.8) | 6 (5.4) | <0.001 |

**Supplementary Table 1. Clinical and laboratory characteristics of children evaluated at pediatric emergency department for non-febrile and febrile seizures.**

For normal distributed variables means ± SDS are shown, while for non-parametric ones median and interquartile range are shown.

*Age range for non-febrile seizures: 0.08–14.8 years, for febrile seizures: 0.50–5.9 years.

^&^Standardized mean differences (SMD): Age=1.40; WBC= -0.61; Neutrophils= -0.72; Platelets= 0.31; Glycemia= -0.40; Serum Na= 1.07; Serum Cl= 0.60; Bicarbonates= 0.86; CRP= -0.76.

*Abbreviations:* AKI, acute kidney injury; Ca, calcium; Cl, chloride; CPK, creatine phosphokinase; CRP, c-reactive protein; EEG, electroencephalography; HC/BC, highest-to-basal creatinine ratio; IQR, interquartile range; K, potassium, Na, sodium; SDS, standard deviation score; WBC, white blood cells.
